# Exploring the Use of Sensors to Measure Behavioral Interactions: An Experimental Evaluation of Using Hand Trajectories

**DOI:** 10.1371/journal.pone.0088080

**Published:** 2014-02-07

**Authors:** Jeroen H. M. Bergmann, Patrick M. Langdon, Ruth E. Mayagoitia, Newton Howard

**Affiliations:** 1 Brain Sciences Foundation, Providence, Rhode Island, United States of America; 2 Centre of Human & Aerospace Physiological Sciences, King’s College London, London, United Kingdom; 3 Department of Engineering, The University of Cambridge, Cambridge, United Kingdom; 4 Division of Health & Social Care Research, King’s College London, London, United Kingdom; 5 Synthetic Intelligence Lab, Massachusetts Institute of Technology, Boston, Massachusetts, United States of America; University of Adelaide, Australia

## Abstract

Humans appear to be sensitive to relative small changes in their surroundings. These changes are often initially perceived as irrelevant, but they can cause significant changes in behavior. However, how exactly people’s behavior changes is often hard to quantify. A reliable and valid tool is needed in order to address such a question, ideally measuring an important point of interaction, such as the hand. Wearable-body-sensor systems can be used to obtain valuable, behavioral information. These systems are particularly useful for assessing functional interactions that occur between the endpoints of the upper limbs and our surroundings. A new method is explored that consists of computing hand position using a wearable sensor system and validating it against a gold standard reference measurement (optical tracking device). Initial outcomes related well to the gold standard measurements (r = 0.81) showing an acceptable average root mean square error of 0.09 meters. Subsequently, the use of this approach was further investigated by measuring differences in motor behavior, in response to a changing environment. Three subjects were asked to perform a water pouring task with three slightly different containers. Wavelet analysis was introduced to assess how motor consistency was affected by these small environmental changes. Results showed that the behavioral motor adjustments to a variable environment could be assessed by applying wavelet coherence techniques. Applying these procedures in everyday life, combined with correct research methodologies, can assist in quantifying how environmental changes can cause alterations in our motor behavior.

## Introduction

The evolutionary development of the hand as part of the upper extremity has been essential for progression of the human race. Bipedalism freed the hands from locomotion for dexterous behavior, such as tool making and communication [1]. Many of the gained advantages of freeing up the hands relate to the interaction of the extremity with objects. It has been suggested that progression in hand function not only provided new ways to fabricate and use tools, but also affected other behavior states. This is illustrated by aggressive behavior, such as clubbing and throwing, that suddenly became available to the early humans because of a change in anatomical design [2]. These findings indicate the range of behaviors that can be influenced by a changing function of the upper extremity. However, performance is not just based on the anatomical properties of the limb, since motor control will define the level of efficiency at which the movements are executed. Movements are precisely controlled by the brain and communication deficits between the musculoskeletal and nervous system lead to direct changes in (motor) behavior. Even at the early stages of life, spontaneous movements differ between premature infants with brain injuries and those without injuries [3]. Motor patterns also alter during our life span and changes are likely to relate to the development of neural mechanisms that underlie the control of the arm and hand [4]. Objective measurements of arm movements could even inform us about associated neurological functioning throughout normal and impaired development. However, they also reveal how behavior changes in response to modest changes in the environment. Both humans and animals seem sensitive to what appears to be only small changes in their surroundings [5], [6]. Yet, we lack the scientific base of how these small everyday alterations might affect our behavior. An accurate tool that quantifies human-object interaction is needed to study this and one potential approach is explored in this paper.

Accurate measurements of human movement during specific tasks can increase the understanding of certain behaviors in response to alterations in our perceived world. Assessment tools need to be able to collect relevant parameters for the duration of a particular activity in order to acquire relevant information regarding the interactions between a person and their surroundings. Traditionally, kinematics and biomechanical aspects of movement are studied with optical motion analysis systems in laboratory settings. Although, this kind of research yields valuable information, the results only remain valid in conditions where no anticipation or reaction to a real-world environment is required [7]. It is preferable to collect data on location during real-life situations where individuals can express “normal” behavior. This kind of data has a higher degree of ecological validity, therefore increasing the external validity of the final results (Locke, 1986). Such an approach would require a portable sensor system that can collect body segment orientation in any environment under a range of different conditions.

Triaxial gyroscopes can be used to measure the angular orientation of a body segment, by integrating the angular velocity signal. However, a relative small offset error of the signal will introduce large integration errors. As the majority of normal human movement generates accelerations below the gravitational acceleration of 9.81 m/s^2^, accelerometers can be used to provide additional inclination information. Since the accelerations that occur are relatively small compared to the gravity vector, the magnitude of the acceleration with respect to gravity can be neglected, thus providing inclination information that can be used to correct the drifted orientation estimate from the gyroscopes. It has been shown that a triaxial accelerometer and gyroscope can be fused together to accurately measure the orientation of human body segments (Luinge & Veltink, 2005). However, this method will be less accurate for movements with relatively large accelerations and it does not provide information of the rotation component around the vertical axis. Further improvements can be made by adding a triaxial magnetometer to the measurement unit. A magnetometer is sensitive to the earth’s magnetic field and gives information about the heading direction. This information can be used to correct for drift of the gyroscope about the vertical axis (Roetenberg et al., 2003). Inertia Measurement Units (IMUs) consisting of a triaxial accelerometer, gyroscope and magnetometer provide consequently the most accurate measurements of angular orientation during movement. IMUs have become more and more popular in the human movement and clinical research field, as they combine certain notable benefits. They are small, portable and lightweight, thereby satisfying the requirements to perform measurements in real-world situations and at the same time providing a cheaper alternative to the laboratory-bound optokinetic systems (Veltink et al., 1996).

The aim of this study is to explore the validity of a wearable sensor system to track the arm (part 1) and investigate if the system can identify how motor behavior changes with small alterations in the environment (part 2). The validity of the sensing device is examined by determining how closely the distal position of the arm (a solid hand and wrist complex) relates to the position acquired by an optical tracking device, during a series of predefined arm movements. Subsequently, wavelet analysis was applied to compare slightly different object constraints within the same everyday task, in order to assess changes in the consistency of the displayed motor behavior. Three different objects, which all have the same functional purpose, were used to introduce changes in the environment.

## Experiments

### Part 1

#### Validation of the measurement system

**Figure 1:**
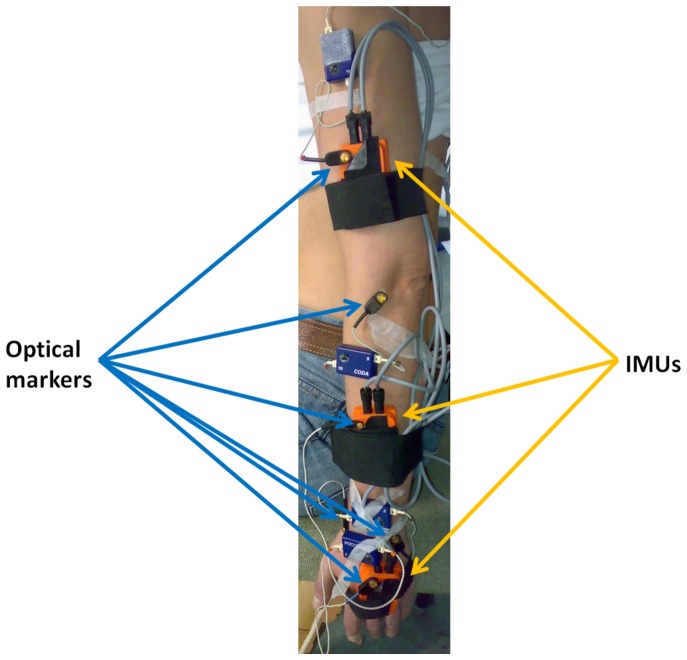
Optical tracking markers and Inertia Measurement Units (IMUs) as attached to the left arm of the participant.

One healthy female participant aged 37 years (height 171 cm, weight 61 kg) volunteered to participate in this study after local college ethical approval (BDM/10/11-12) from the *King’s College London* College Research Ethics Committee and written informed consent was obtained for all parts of this study. Three IMUs (MTx, Xsens Technologies B. V., Enschede, Netherlands) were placed on the hand, lower and upper arm (Figure 1). Straps were used to provide a preloading force and thereby decreasing measurement errors [8]. The sensors (30 g each) were securely attached to each body segment in order to ensure that the orientation of the sensor with respect to the body segment did not change.

The placement of the sensor determined the relationship of the sensor axis to the anatomical coordinate system, as the sensor coordinate system was fixed to the device. The Z-axis of each IMU coordinate system was physically placed to run as close as possible perpendicular to the sagittal plane, while at the same time minimizing relative motion between sensors and underlying bones. As the participant sat as still as possible with the arm hanging to the side of the body, a further analytical correction was applied by software (MT Software V2.8.1, Xsens Technologies B. V., Enschede, Netherlands). The alignment program placed the Z-axis of each IMU in line with gravity (vertical plane) with the new X-axis of all the sensors perpendicular to the Z-axis and along the line of the original global X-axis, while the Y-axis was chosen as to obtain a right handed coordinate frame. The non-orthogonality between the axes of the body-fixed coordinate system was less than 0.1°.

Active Codamotion markers (Codamotion, Charnwood Dynamics, Leicestershire, UK) were placed, using double-sided adhesive tape, on the radial styloid process, ulnar styloid process, lateral epicondyle, medial epicondyle, acromion, spinous process of the seventh cervical vertebra and the IMUs (Figure 1). A standard system configuration was used for data acquisition by the Codamotion and Motion Tracker software. The cameras of the optical tracking device were positioned in such a way that the position data of the right side could always be obtained during movement. The three dimensional (3D) position of these markers can be determined with an accuracy of ±1 mm [9].

Data for both the Codamotion and the IMUs was acquired at 100 Hz and an electronic pulse was used to synchronize the two measurement systems. All further data analysis was done using Matlab (MathWorks, Inc., Natick, MA, USA).

#### Motion sequences

**Figure 2:**
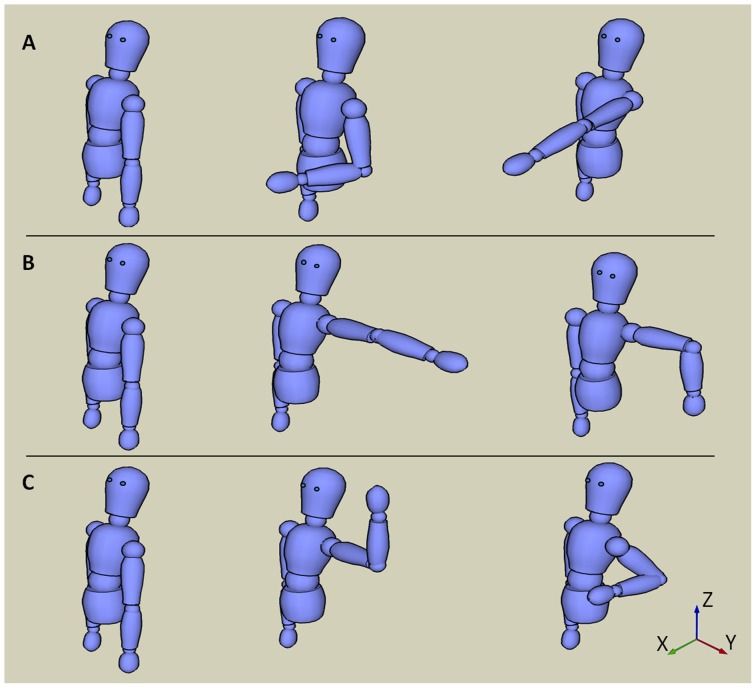
Arm positions used. Each arm position sequence used is identified by a letter (A, B or C) and consisted of three succeeding positions the participant was instructed to attain and then hold for approximately 10 s.

The participant sat on a chair with the arm rested at the side of the trunk. The subject was asked to perform three different sequences each consisting of three different positions (Figure 2) and each held for roughly 10 seconds. The first sequence started with the arm fully extended, hanging by the side, in the start position, followed by flexing the elbow to 90° and keeping the forearm in neutral, after which the participant was asked to move to 90° of shoulder anteflexion, with the arm fully straight and pointing forward (sequence A). In the second sequence, the participant began in the same starting position as the first sequence and was then instructed to move to 90° of shoulder abduction with the elbow fully extended, from this position the elbow was flexed to 90° and an internal rotation was performed (sequence B). The last sequence also had the same starting position as the previous two, from which the participant moved her arm to 90° of shoulder abduction and 90° elbow flexion with an external rotation; this was followed by moving to 45° retroflexion in the shoulder and 120° of flexion in the elbow (sequence C).

#### Biomechanical model used with the IMU data

**Figure 3:**
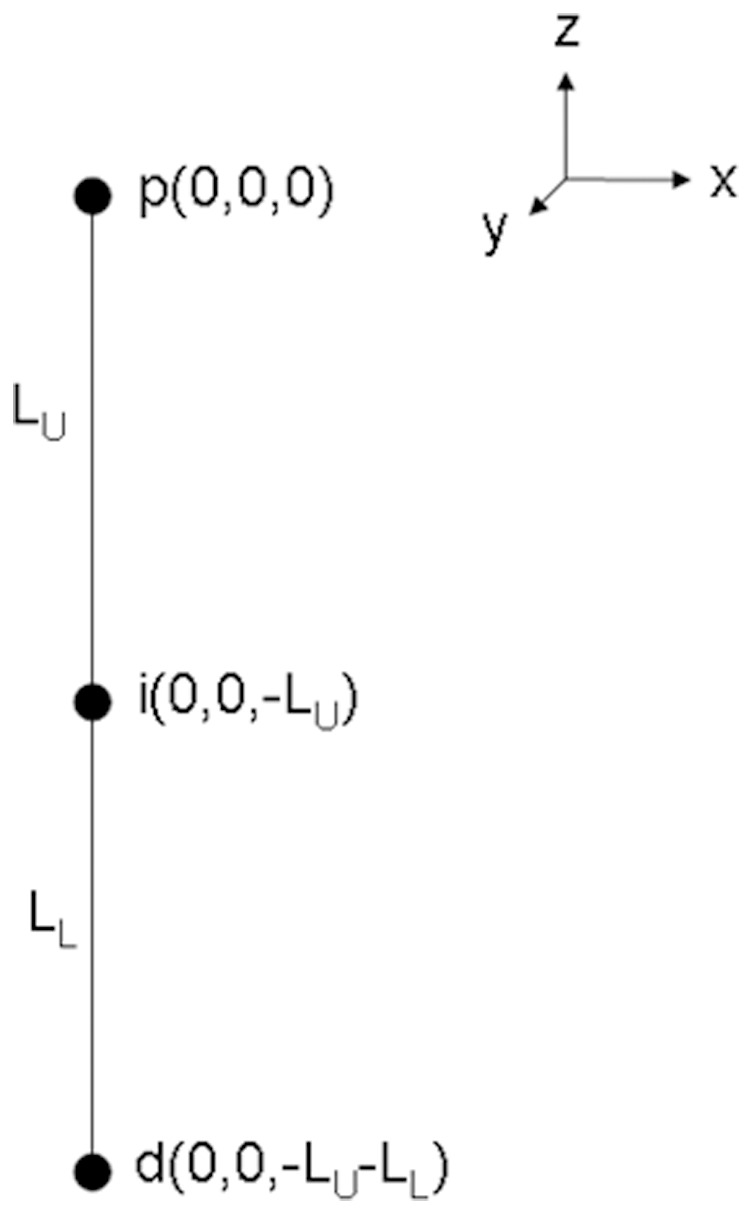
Initial condition of the two-link model. Segment lengths were taken from anthropometric data [9]. The proximal point (**p**) represents the shoulder; the intermediate point (**i**) is the elbow and the distal point (**d**) the hand. All positions are given in (X,Y,Z). (LU) length of the upper arm; (LL) length of the lower arm and hand.

The upper extremity can be approximated as a multilink chain, with each body part as a rigid segment and its movement represented by one IMU [10]. A simplified two-segment 3D model was used that only consisted of two (upper and lower arm) rigid segments (Figure 3). The shoulder blade (scapula) movement was not taken into account. In addition, the hand and wrist were considered as a single rigid segment for ease of application of the model. The upper arm movement was essential, as it has already been shown that functional changes of the hand-wrist complex directly affects movement patterns at the shoulder [11]. To keep the model as simple as possible only the signals from the sensors that were absolutely vital to reproduce the general movement pattern were selected. Body segment lengths were calculated from anthropometric data [10], as a percentage of the body height of the participant.

#### Analysis

The 3D representations of the distal point of the left arm (point [d] in figure 3) obtained using the two measurement devices were compared by calculating a two-tailed Pearson product-moment correlation coefficient (r) and by calculating the root mean square error (RMSE) between the two signals [7], [12], [13]. The dynamic range, defined as the largest possible signal (full range of motion) divided by the smallest possible signal [14] (maximum error), was calculated for the IMU based model. Independent analysis of each direction of movement, referred to as X, Y and Z, was performed. In addition, the Euclidean norm 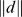 was determined by
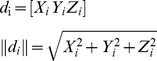
with *d_i_* representing a vector based on *X_i_*, *Y_i_* and *Z_i_* with index point *i*.

#### Results

**Figure 4:**
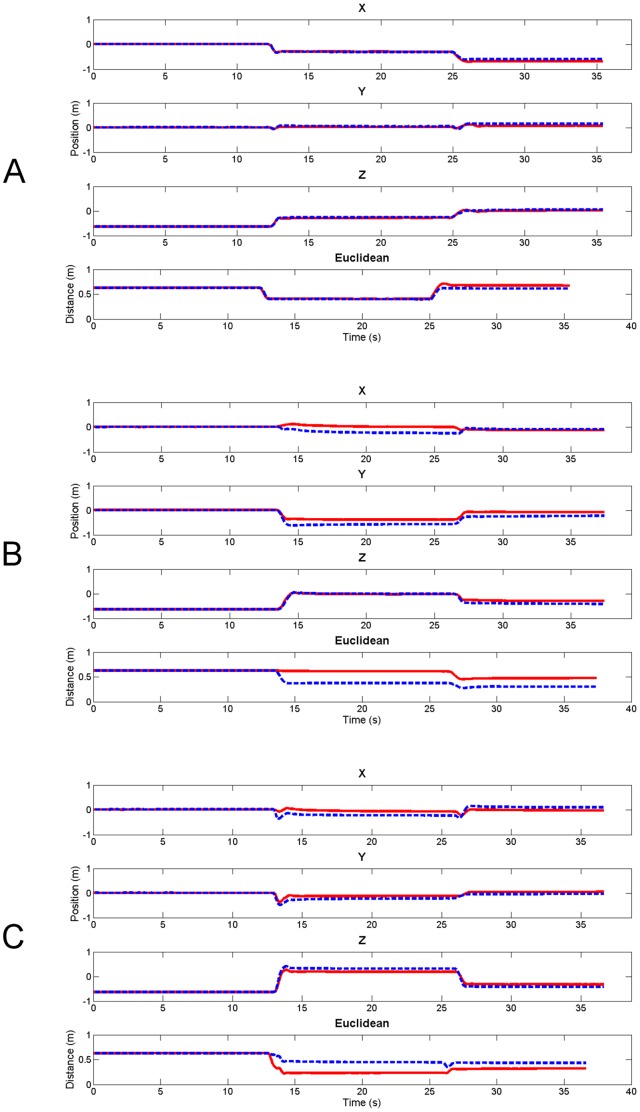
Positions of the hand in each direction (X, Y, Z and Euclidean norm) and for every sequence. Dashed blue lines are the positions obtained from the optical tracking device and the solid red lines correspond to hand positions calculated by the biomechanical model using IMU data.

The shape of the curve describing the movement was on the whole highly comparable between the two measurement methods (Figure 4). High Pearson correlation coefficients were found in both the Y and Z direction for all movement sequences (Table 1). After an initial high correlation in the X direction for sequence A, a drop in correlation occurred for the subsequent sequence. The movement of the hand in the X direction was poorly correlated in sequence B. However, the RMSE calculated in the X direction for this sequence was not higher than those found for sequence A and C. The best accuracy was found for sequence A, with RMSE ranging from 0.02 to 0.05 meters. Sequence B not only had the poorest correlation between the methods, it also suffered from the highest RMSE. The Euclidean norm showed a lower correlation for sequence B, but had very strong correlations for both A and C. The highest dynamic range was found for sequence B in direction X. In general, the Z direction performed the best in terms of highest correlations and lowest RMSEs. It also had relative high dynamic ranges across all sequences.

**Table 1:** Pearson correlation coefficients, Root Mean Square Errors and dynamic range between the positions obtained by both methods.

	Pearson correlation coefficient (p<0.01)			Root Mean Square Error (meters)			Dynamic Range		
S	*A*	*B*	*C*	*A*	*B*	*C*	*A*	*B*	*C*
**X**	0.99	−0.11	0.60	0.04	0.14	0.13	50.46	0.95	0.97
**Y**	0.95	0.97	0.93	0.05	0.15	0.08	3.46	2.38	2.10
**Z**	0.99	0.98	0.99	0.02	0.07	0.10	44.76	7.35	9.48
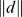	0.98	0.73	0.95	0.03	0.17	0.15	7.0	2.07	1.3

S stands for motion sequence; X, Y and Z represent the directions of movement for the upper limb point and the Euclidean norm is given by 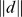.

#### Discussion

Single plane movements (e.g. sequence A) seem to provide the best correlations between methods. However, even in more complicated movement patterns (sequence B and C) motion of the distal part of the upper limb model relates well to the motion of the optical tracking marker in both the Y and Z direction. The low negative correlation found in sequence B does not affect the overall movement pattern too much, as the hand position changed more for the Y and Z direction (Δ*_Y_* = 0.39 and Δ*_Z_* = 0.68 m) than for the X direction (Δ*_X_* = 0.25 m).

The Euclidian norm provides a method for dimensionality reduction and the results indicate it performed relatively well across the explored sequences. On the whole, the principal movement patterns can be picked up by the proposed method based on body-worn sensors, but they do not provide high absolute accuracy of the position of endpoints. The dynamic range showed that information could be extracted from the dominant planes of motion. Yet, smaller deviations, particular in the X direction, did (almost) not register beyond the noise level. The proposed method can best be applied to movement patterns, with large changes in positions. The proposed model is most suitable for motor behavior that involves large ranges of motion at the shoulder complex.

Although motion artifacts do have a detrimental effect on outcome, the largest source of accuracy errors is more likely due to the fact that the biomechanical arm model was based on only two segments. It has been shown that IMUs can obtain accurate estimations of arm position when applied during movement patterns that require a very limited range of motion [15]. The sequences in this study were specifically selected in order to obtain an insight into the accuracy of the proposed model over a fuller range of arm movements. Also, the difference found between the two methods could be contributed to inaccuracies associated with the relative movement of the sensors or markers compared to the underlying bones. Due to this relative movement between the optical tracking marker and the underlying bony landmark, artifacts in position data can occur [16]. Displacements of more than 20 mm between skin and underlying bone have been reported for optical tracking systems [17].

The presented validation results relate to the agreement of the two systems within this study. These outcomes do not reflect validation of this system for an out of experiment population and are meant to provide the required internal validation that is needed for further exploration of the method within this paper.

### Part 2

#### Analyzing changes in motor behavior

**Figure 5:**
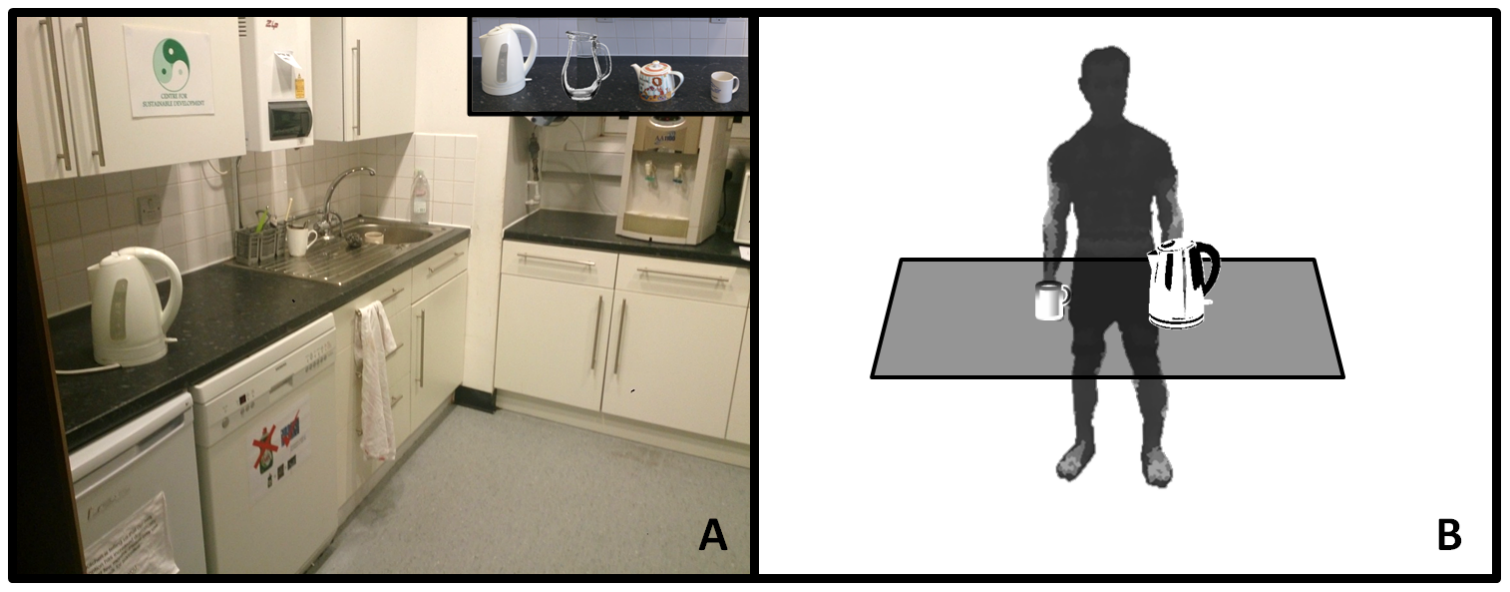
Experimental setup. **A** Picture of the kitchen that was used with at the top right corner an inset of the containers and mug **B** Schematic of the experimental setup that was applied.

The upper limb model was subsequently tested by having three healthy subjects (2 males and 1 female, aged between 27 and 42 years), using three different liquid container designs (pitcher, teapot and kettle). From an initial starting position (hands placed along their side), participants were asked to pick up the container with liquid with their left hand and pour a little bit into a cup without spilling. All subjects confirmed that they were accustomed to pouring with their left arm and the sensor system was fitted on that side. Both the container and cup were placed in a preset location to ensure agreement between subjects and tasks. Apart from these basic constrains, participants were free to choose their own preferred movement path and speed. Subjects were asked to repeat the pouring task 8 times during two separate trials. The start and end position consisted of the arm resting on the side of body. The measurements were taken in the kitchen where the subjects would normally prepare their drinks (Figure 5). The container and mug were placed about 20 cm away from the edge of the kitchen countertop.

**Figure 6:**
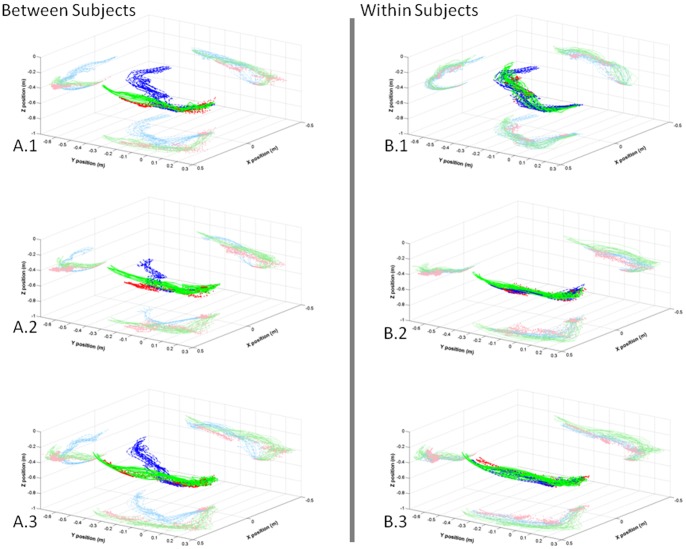
Traces of the hand computed using a two-linked segmental model. All figures starting with **A** compare the patterns between three subjects (blue, red and green) interacting with a pitcher (**A.1**), teapot (**A.2**) and kettle (**A.3**). The figures labeled with a **B** show the traces for each subject (**B.1**, **B.2** and **B.3**) using pitcher (blue), teapot (red) and kettle (green). All plots show a 2D projection of the data for each plane.

The purpose of these measurements was to show the utility of the sensing system for determining coherence in motor behavior related to the individual or object (container). The previous mentioned local college ethical approval also covered this part of the study and written informed consent was obtained from all three subjects. Observation of the initial results shows that the difference is more profound, between subjects than within subjects (Figure 6).

#### Analysis

The data presented in Figure 6 can be compared using a range of methods. As the signal varies in time, one could apply a Fourier analysis that relies on adding together the appropriate infinite sum of sine waves. However, most behavioral signals are finite and require the detection of localized features. In this case the use of wavelets is more appropriate. Fourier analysis is based on a single function that is scaled, but the wavelet transform also shifts the function, generating a more accurate description of the signal [18]. A wavelet is a special case of a vector in a separable Hilbert space that generates a basis under the action of a collection of unitary operators defined in terms of translation and dilation operations [19]. We use a continuous wavelet transform (CWT) to divide the signal into wavelets, allowing us to analyze the frequency content over time. This information can then be used to compare two signals and find a potential relationship between them. Regions where the signals have equal power or phase behavior indicate an association. The wavelet coherence can be interpreted, to some extent, as a measure of local correlation [20]. Coherence measures the variability of time differences between two time series in a specific frequency band [21]. It can be expected that the variance for a particular behavioral task (e.g. pouring) is somewhat comparable across repetitions performed by the same subject who is using the same container. Changing the situation, in our case by applying a different container, can cause interference in a “similar” set of motor behaviors [22]. Therefore, wavelet-coherence is initially computed only within repeated movements of one subject and one container. A subsequent comparison is made between the summed results of each of the subjects and objects.

A Morlet waveform was selected for the wavelet analysis, as it was expected to show an appropriate match with the performed activities [23]. The wavelet coherence of two time series *x* and *y* can be described as,
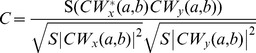
where S is the smoothing operator, while CW_x_ *(a,b)* and CW_y_ *(a,b)* denote the continuous wavelet transforms of the signal *x* and signal *y* at the scales *a* and the positions *b* [24]. A good practical introduction to wavelet coherence can be found in [25]. The wavelet coherence can be used in this experiment to compare signals between subjects or between containers (objects). Data were analyzed using the wavelet toolbox in Matlab (MathWorks, Inc., Natick, MA, USA).

**Figure 7:**
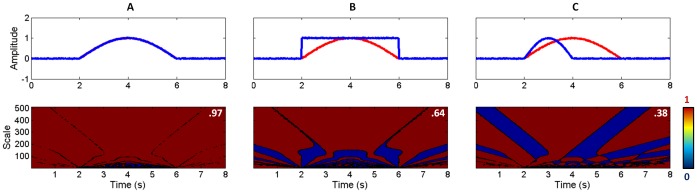
Example of how the wavelet coherence changes over three samples of wave patterns. Zero-mean Gaussian noise is added to all signals. Top plots: **A** Red signal shows a sine wave with a frequency *f* and the blue trace has a frequency of 1.001*f*. **B** Red signal shows a sine wave, while blue is a Haar wave with the same frequency *f.* **C** The red signal shows a sine wave with a frequency *f* and the blue trace has a frequency of 2*f*. **Bottom plots** show the wavelet coherence for each example. The heat map displayed on the right side specifies the coherence. The mean wavelet coherence value (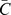) is displayed in the corner of each bottom plot.

Examples of simulated outcomes for wavelet coherence are given in Figure 7 in order to provide some further background to the reader. The examples show the wavelet coherence between different sine and Haar waves. The first example (A) shows the outcome between two almost identical sine waves. The second example (B) shows a Haar and sine wave with the exact same frequency. In the last example (C) there is a factor 2 difference in frequency, between the two waves. It is clear from Figure 7 that the localized similarities differ depending on the signals that are compared. The amount of divergence can subsequently be described, as an average wavelet coherence value (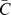). This value is simply calculated by first averaging across the scales at each time point (columns) and subsequently across the time points themselves,
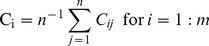


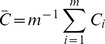
with *C* representing the coherence with rows *i* and columns *j* for lengths *n* and *m*. High scales are associated with low frequencies, while the low scales portray the high frequencies. The high scales (low frequencies) are of particular interest, as everyday living activities normally take several seconds to complete and even longer when restricted by impairments [26].

The subjects poured several times with each container providing the opportunity to calculate the mean wavelet coherence of the Euclidean Norm across all possible pairwise repetitions. Based on these outcomes a between and within subject 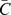 could be computed by taking the average of all subjects or objects. This provided a simple measure of consistency in motor behavior, which was used to explore the notion that consistency in motor behavior might differ between subjects and containers.

#### Results

**Figure 8:**
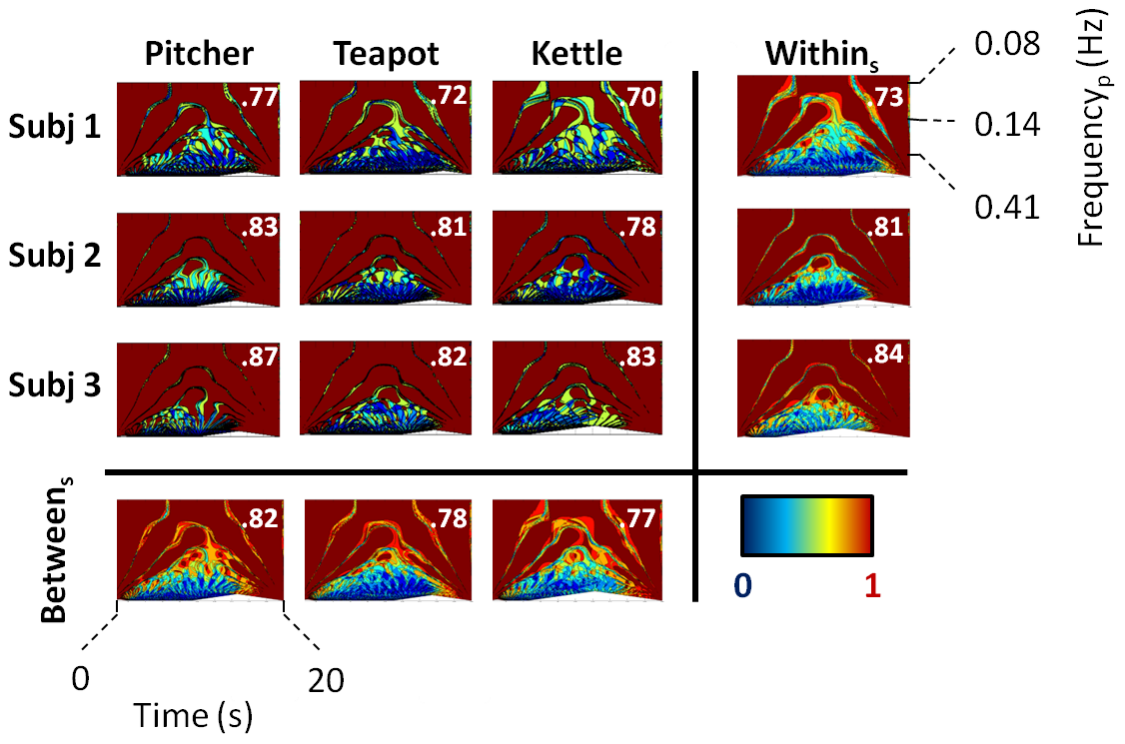
Wavelet coherence plots of the Euclidean norm. The mean coherence across 8 movement repetitions is displayed for each subject, while using one of the three containers. At the end of the rows all wavelet coherences per subject are averaged (Within_s_). At the bottom of each column all subjects are averaged for each container (Between_s_). The warmer the color of a region, the greater the coherence is between the two signals. The full wavelet coherence is subsequently averaged for each subject and container to generate a single value (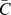) that is displayed in top corner of each plot.

The average (± standard deviation) duration of the pouring tasks was 10.6 (±2.1) seconds with a range of 7.3 to 16.8 seconds. The coherence results are provided in Figure 8. Inspection of the wavelet coherence plots show that subject 1 has a distinctive different outcome compared to subject 2 and 3. Subject 1 also showed the highest overall phase difference (.73) with respect to time and scale. Overall the pitcher yielded the highest 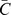 across subjects (.82), while the kettle had the lowest (.77). This example shows the ability of the analysis method to differentiate between movement patterns. The difference in localized features indicates how much and when consistency in motor behavior differs within subjects and between objects (Figure 8).

#### Discussion

This preliminary dataset demonstrates the possible utility of the simplified upper limb model in a real-life setting, by combining concepts such as wavelets and unitary math. The results showed that wavelet analysis can be applied to compare everyday movement tasks, such as pouring. The wavelet coherence estimates the association between two signals with respect to both time and scale. The outcomes obtained in the wavelet coherence analysis focused on how consistent motor behavior was within and between subjects. It became clear that subject 1 was the least consistent in motor behavior, which potentially relates to the selection of an alternative motion path (Figure 6. A) compared to subjects 2 and 3. The pitcher also seemed to provide a context in which each subject was able to display more consistent behavior. The example explored in this study has limited generalizability, due to the small experimental sample size and the focus on the left hand. The aim of this study was not to provide results that could be easily extrapolated, but to show that behavioral consistency in activities of daily living could be explored in more detail using sensors and wavelets.

The importance of situations for determining behavior is well-known and cross-situational consistency of behavior has already been associated with how much situations are “alike” [27]. Higher measurement resolutions could provide a richer understanding of behaviors that are assumed similar. It even allows for possible detection of minor environmental changes, such as a slightly different interaction object, that are now often overlooked.

Defining behavior within the field of behavioral science has led to many different interpretations and opinions [28]. A recent study generated a more evidence based definition that stated that behavior is the internally coordinated responses (actions or inactions) of whole living organisms (individuals or groups) to internal and/or external stimuli, excluding responses more easily understood as developmental changes [28]. The majority of these responses reflect coordinated actions of the human musculoskeletal system, despite the fact that these actions are emerging properties of multiple attributes.

The focus on real-world repeatability of motor behavior comes from one of the most cited articles in cross-species behavior [29]. The authors found that repeatability estimates were higher in the field compared to the laboratory and repeatability was higher when the interval between observations was short. Although, humans are likely to differ from other species these findings offer an interesting argument to collect more real-world data. There is also evidence that repeatability increases with human ageing and this has been linked to the process of consolidated identity or reputation [30], [31]. The proposed method might provide a new way of exploring real-world behavior with a system that is not limited to a specific setting. Although, camera tracking with consumer products has now been shown to be useful for low-cost hand tracking [32], such a method still remains restricted to tracking people within a limited field of view. In addition, optical tracking systems will lose their utility once this field is obstructed.

## Discussion

### Limitations of the Study, Open Questions, and Future Work

The Euclidean norm was utilized for data analysis, as it represents a simple magnitude value. Caution needs to be taken with only applying the norm as parameter, as the dimensional reduction and consequent loss of information might only be appropriate for certain hypotheses. Nonetheless, the method introduced in this paper could also take in other sensing variables. In principle, questions regarding how specific situations can affect consistency in behavioral control of the arm could be investigated using the proposed method.

As mentioned previously, the small subject sample minimizes any generalizability of the presented results. Small sample sizes have been used to pilot applications for body-worn sensors [33], but it comes with the limitation that further research needs to be done in order to establish the boundaries of the external validity of the proposed system and analysis method. Although, the coherence approach should provide relevant outcomes with only a few trials [34], it is recommended not to extrapolate these results beyond the explorative nature of this study.

Recently, it has been argued that more progression is need in the conceptualization and measurement of situations to better understand personality and the ongoing person–situation debate [33]. The presented work here explored a measurement tool that could be applied for studying person–situation paradigms. A larger study is required to determine if the system can be used to determine how consistency in everyday living depends on concepts such as personality.

Future work should focus on an even less intrusive version of this device, as it has been shown that utility might be affected by the measurement tool itself if it is not fully unobtrusive [35]. Integrating the sensor system into a garment is a potential adaptation that would make the system less noticeable, subsequently generating data sets that even more closely represent real-life behavior. Furthermore, one can also start applying unitary mathematics directly to predict human interaction by modifying recently presented methods for predicting linguistics [36]. Such analysis needs to be applied to larger datasets in order to disentangle natural variation from variability that arises due to a changing environment. Comparing the obtained outcomes to a reference database would provide a method to track changes over time and differentiate between environmental changes and natural variability. The methodology can potentially be developed into a long-term tracking system that identifies how people interact in everyday life, thus providing a possible continued data stream for investigating behavioral consistency of an individual during ever changing natural surroundings. This approach might be interesting for a range of different research fields, including product design and human factors.

## Conclusion

The current tool focuses on measuring aspects of arm movement, which represents only a small part of overall human behavior. However, the results obtained with the presented system have shown how specific sensor modalities could be used to provide feedback regarding behavioral changes in response to a changing (object-related) environment. The example given in this paper shows how new sensing tools can help evaluate behavior and potentially improve our understanding of our interaction with our environment. The concept of using body-worn sensors to gather behavioral information in itself is not new. Applying sensor systems to measure animal behavior has been important for understanding how animals interact with their environment and is one of the fundamental aims of animal ecology [37]. Moreover, human behavior has also been widely tracked using sensors [38], [39]. Yet, often this monitoring relates to energy expenditure or activity recognition and those studies that do track upper limb movement have frequently a purely clinical aim.

Recently, patients with and without the behavioral variant of frontotemporal dementia have been identified as similar in a caregiver-based assessment of activities of daily living, whereas a clear distinction was identified with a performance based measurement [40]. This example highlights the need to quantify (motor) behavior beyond the level that is often applied. Small changes in our environment are not often taken into account, while they do influence our behavior. For example, it is known that changing colors and shapes directly alters behavior [41], [42]. We propose here that wearable sensor systems can be utilized to understand how small changes in a real-life environment affect us. This approach combined with well-developed research protocols could help us better quantify how our interactions are affected by our everyday surroundings.

## Supporting Information

Data S1 The supporting information contains the Euclidean norm (m/s^2^) for each subject and container condition described in part 2. Only data that crossed the threshold value of.625 was defined as movement. All other values were set to the aforementioned threshold value to minimize their impact in the wavelet analysis. All starting points of the movements were aligned.
(XLS)
